# Thyroid Profile in the First Three Months after Starting Treatment in Children with Newly Diagnosed Cancer

**DOI:** 10.3390/cancers15051500

**Published:** 2023-02-27

**Authors:** Chantal A. Lebbink, Cor van den Bos, Miranda P. Dierselhuis, Marta Fiocco, Annemarie A. Verrijn Stuart, Eef G. W. M. Lentjes, Sabine L. A. Plasschaert, Wim J. E. Tissing, Hanneke M. van Santen

**Affiliations:** 1 Department of Pediatric Endocrinology, Wilhelmina Children’s Hospital, University Medical Center Utrecht, 3584 CX Utrecht, The Netherlands; 2 Princess Máxima Center for Pediatric Oncology, 3584 CS Utrecht, The Netherlands; 3 Mathematical Institute Leiden University, The Netherlands and Department of Biomedical Science, Section Medical Statistics, Leiden University Medical Center, 2333 ZA Leiden, The Netherlands; 4 Laboratory Clinical Chemistry and Hematology, University Medical Center Utrecht, 3584 CX Utrecht, The Netherlands; 5 Department of Pediatric Oncology, University of Groningen, University Medical Center Groningen, 9713 GZ Groningen, The Netherlands

**Keywords:** thyroid dysfunction, childhood cancer treatment, pediatrics

## Abstract

**Simple Summary:**

Thyroid dysfunction during childhood may affect daily energy, growth, body mass index and bone development. Thyroid dysfunction may occur in children with cancer due to chemotherapy or other drugs, radiotherapy, the tumor itself or severe illness. The aim of this prospective study was to determine the percentage, severity and risk factors of changing thyroid hormone concentrations in the first three months of childhood cancer treatment. Subclinical hypothyroidism (normal thyroid hormones, with elevated thyroid stimulating hormone (TSH) according to age) was present in 8.2% of children at diagnosis and 2.9% of children three months after starting treatment. Subclinical hyperthyroidism (normal thyroid hormones, with lowered TSH values according to age) was present in 3.6% of children at diagnosis and 0.7% of children after three months. In 28% of children, the concentration of free thyroid hormone (FT4) decreased by ≥20%. We conclude that children with cancer are at low risk of developing hypo- or hyperthyroidism in the first three months after starting treatment but may develop a decline in FT4.

**Abstract:**

Background: Thyroid hormone anomalies during childhood might affect neurological development, school performance and quality of life, as well as daily energy, growth, body mass index and bone development. Thyroid dysfunction (hypo- or hyperthyroidism) may occur during childhood cancer treatment, although its prevalence is unknown. The thyroid profile may also change as a form of adaptation during illness, which is called euthyroid sick syndrome (ESS). In children with central hypothyroidism, a decline in FT4 of >20% has been shown to be clinically relevant. We aimed to quantify the percentage, severity and risk factors of a changing thyroid profile in the first three months of childhood cancer treatment. Methods: In 284 children with newly diagnosed cancer, a prospective evaluation of the thyroid profile was performed at diagnosis and three months after starting treatment. Results: Subclinical hypothyroidism was found in 8.2% and 2.9% of children and subclinical hyperthyroidism in 3.6% and in 0.7% of children at diagnosis and after three months, respectively. ESS was present in 1.5% of children after three months. In 28% of children, FT4 concentration decreased by ≥20%. Conclusions: Children with cancer are at low risk of developing hypo- or hyperthyroidism in the first three months after starting treatment but may develop a significant decline in FT4 concentrations. Future studies are needed to investigate the clinical consequences thereof.

## 1. Introduction

Thyroid hormones are essential during childhood for adequate mental development, linear growth, bone development and metabolic regulation [1,2]. Signs and symptoms of thyroid dysfunction can be overweight, declining linear growth, mental retardation in the young, constipation (hypothyroidism), tachycardia and growth acceleration (hyperthyroidism), or fatigue and emotional imbalances (both). In children with cancer, thyroid dysfunction may present with symptoms that are regularly observed during childhood cancer treatment and thus may be overlooked.

The thyroid gland can be damaged in children with any type of cancer by the tumor itself, chemotherapy (e.g., busulphan), radiation exposure or immunotherapy, resulting in thyroidal hypo- or hyperthyroidism [3]. In several small studies, the prevalence of primary hypothyroidism during cancer treatment varied between 0 and 18% [4,5,6,7,8,9]. Next to damage of the thyroid gland, thyroid hormone metabolism in children with cancer may also be distorted due to damage of the hypothalamic–pituitary region as a consequence of a brain tumor or cranial irradiation (central hypothyroidism). Moreover, specific drugs may influence the thyroid profile without actual thyroid or pituitary gland damage, as is seen, for example, after the administration of asparaginase with a decrease in thyroxine binding globulin (TBG) concentration [8] or after the administration of corticosteroids with lowered thyroid stimulating hormone (TSH), triiodothyronine (T3) and TBG concentrations and increased reverse T3 (rT3) concentrations [8,9].

Lastly, thyroid hormone metabolism may change during childhood cancer treatment as a consequence of an adaptive mechanism during illness called ”euthyroid sick syndrome” (ESS) [10]. In this case, concentrations of thyroxine (T4) and T3 decrease due to two mechanisms, (1) downregulation of hypothalamic thyrotropin-releasing hormone (TRH) secretion and (2) changed activity of the liver deiodinases, resulting in decreased conversion of T4 into T3 and increased conversion of T4 into rT3 [11]. In children, EES has been described during severe illness and anorexia and is thus not associated with the underlying disease per se, but with its severity [12]. For the presence of ESS, different definitions are used, and in the few small studies that have been conducted, the prevalence of ESS during childhood cancer treatment, depending on its definition, varied between 0 and 100% [4,5,6,7,8,9]. When children with cancer have hypo- or hyperthyroidism due to pituitary or thyroidal damage, this is considered a pathophysiological state and needs treatment. However, in case of acute illness, changes in the thyroid profile (ESS) are considered “physiological” and may even be protective. Therefore, it is not recommended to treat children who develop low thyroid hormone concentration during acute illness with thyroid hormone [13]. 

In children who develop mild central hypothyroidism after treatment for a brain tumor, a decline in FT4 of >20%, even within reference ranges, was shown to be clinically relevant [14]. Although mild central hypothyroidism may not be comparable with ESS, it may be hypothesized that a prolonged decline in the FT4 concentration of >20% in children who are not acutely but “chronically” ill (such as during a two-year treatment period for childhood leukemia) does impact bone, muscle and body mass index (BMI) development or daily energy [15]. This has not been studied thus far. Because there is lack of studies reporting on thyroid hormone metabolism in large cohorts of children treated with cancer, we aimed to evaluate the percentage, severity and risk factors of a changed thyroid profile in children during treatment for cancer. 

## 2. Methods and Patients

### 2.1. Patients

We performed a prospective observational cohort during a two-year period (January 2020 to December 2021). The thyroid profile was measured at diagnosis and three months after starting chemotherapy or radiotherapy in newly diagnosed children (<21 years) with leukemia, lymphoma, sarcoma or a non-pituitary brain tumor at Princess Máxima Center for Pediatric Oncology. Children with known previous thyroid disease, Down syndrome, a thyroid cancer predisposition syndrome, a history of neck irradiation or meta-iodobenzylguanidine (MIBG) treatment, or a brain tumor in the hypothalamic–pituitary region were excluded. 

### 2.2. Data Collection

The thyroid profile, using TSH, FT4 and rT3, was measured at the time of diagnosis (range of ±35 days from diagnosis) and three months later (range of 60–160 days after diagnosis). Anti-thyreoperoxidase (anti-TPO) concentrations were measured at diagnosis. Blood results were interpreted by the treating physician. In case of aberrant thyroid function tests (FT4 < or > reference range or TSH <0.30 or >10 mU/L) children were referred to the pediatric endocrinologist and treated if needed.

Clinical data on anthropometrics (height, weight and BMI), general well-being (body temperature, vomiting and nutritional status) and overall physical condition were extracted from patients’ electronic medical records on the day of blood sampling. Physical condition was scored as “good” (no complaints), “medium” (moderate complaints, “not feeling well” or “feeling tired”) or “poor” (severe complaints or “feeling ill”) as reported by the health care provider in the electronic patient chart.

### 2.3. Laboratory Assays 

A description of the laboratory assays is shown in Supplementary File S1. 

### 2.4. Definitions 

Thyroidal hypothyroidism was defined as present if the plasma TSH concentration was above the reference range (5.0 mU/L), combined with a plasma FT4 concentration below the reference range. Thyroidal subclinical hypothyroidism was defined as present if the plasma TSH concentration was above the reference range (5.0 mU/L), combined with a plasma FT4 concentration within the reference range. Subclinical hyperthyroidism was defined as present if the plasma TSH concentration was below the reference range (5.0 mU/L), combined with a plasma FT4 concentration within the reference range. Central hypothyroidism was defined as present if the plasma FT4 concentration was below the reference range, combined with non-elevated TSH concentration in combination with non-elevated rT3 concentration. ESS was defined as present if the plasma FT4 concentration was below the reference range, combined with a non-elevated TSH concentration in combination with an elevated rT3 concentration. 

### 2.5. Statistics

Data are presented as means ±SDs or medians (ranges) for continuous data variables, depending on the distribution. Data are presented as percentages for categorical variables. Differences between groups were examined using unpaired Student’s *t*-tests for normally distributed continuous data and Mann–Whitney U tests for continuous data with a skewed distribution. For categorical data, χ^2^ tests or Fisher’s exact tests (if the assumptions for chi-square were violated) were used. Between-time-point differences were evaluated using paired Student’s *t*-test for continuous data with a normal distribution and Wilcoxon matched-pair signed rank test for continuous data with a skewed distribution. To assess the violation of normality distribution, QQ plots of the residuals and the Shapiro–Wilk test were used. For statistical analysis of changes in thyroid hormone concentrations, only paired blood samples per patient were used. The Pearson correlation coefficient was estimated to study the strength of linear associations between two continuous variables.

Multivariable logistic regression analyses were used to estimate the association between covariates and two outcomes: elevated rT3 concentrations and ≥20% decline in FT4 concentrations. Independent variables included in the multivariable logistic regression were selected by estimating the univariate model and by considering the clinical relevance of each variable. Therefore, in the final regression model, not only variables that were significant in the univariate analysis were included, but also factors that were clinically relevant. Odds ratios (ORs) along with 95% CIs are reported. Analyses were performed using SPSS, version 27.0. *p*-values of <0.05 were considered statistically significant.

### 2.6. Ethics

The research protocol was approved by the medical ethical committee of Princess Máxima Center (NedMec NL69960.041.19). For ethical reasons, blood samples for the study were only taken if sampling for clinical reasons was simultaneously performed. Informed consent was given by all children and/or their parents/legal representatives depending on age.

## 3. Results

### 3.1. General Patient Characteristics

**Figure 1:**
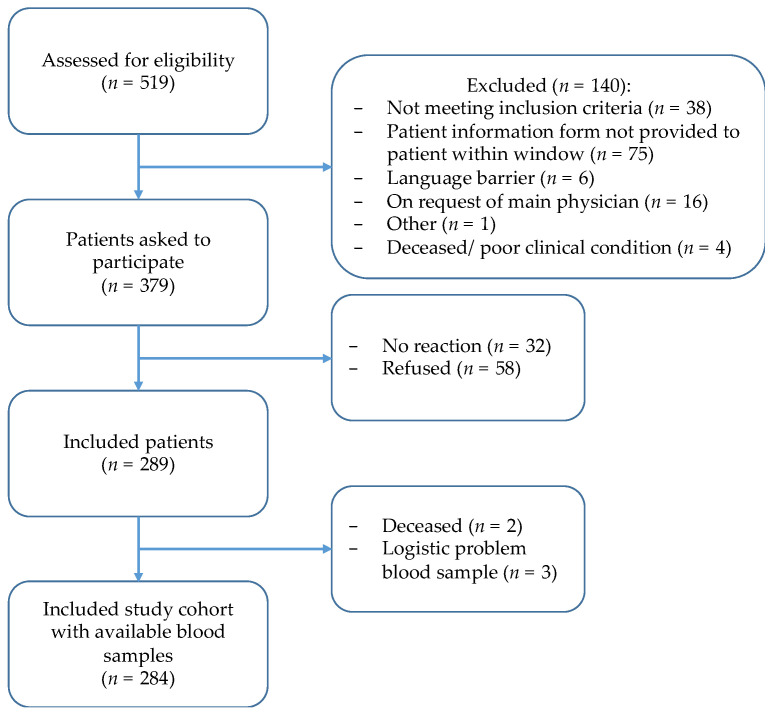
Inclusion flowchart of THYRO-Dynamics study.

Of 519 children assessed for eligibility, 284 were included (Figure 1). Of the included children, 141 (50%) were diagnosed with leukemia, 74 (26%) with lymphoma, 38 (13%) with sarcoma and 31 (11%) with a brain tumor (Table 1). The median age at diagnosis was 9.4 years (range of 0.0–19 years), and 127/284 (45%) children were female.

### 3.2. Thyroid Profile

At diagnosis, TSH and FT4 were both measured in 220 children, in 81% (179/220) of which, both were within reference ranges (Table 2). Three months after diagnosis, in 91% (252/276) of children, both TSH and FT4 concentrations were found to be within reference ranges. In two children (1.2%), elevated anti-TPO antibodies were detected, and both were euthyroid.

#### 3.2.1. (Subclinical) Hypo- and Hyperthyroidism

At diagnosis, 8.2% (18/220) of children had subclinical hypothyroidism with a median TSH concentration of 6.30 mIU/L (range of 5.00–11.00). In 3.6% (8/220) of children, subclinical hyperthyroidism was found (median TSH of 0.21 mIU/L (range of 0.07–0.34)).

Three months after diagnosis, 2.9% (8/276) of children had subclinical hypothyroidism (median TSH of 6.75 mIU/L (range of 5.30–11.00)). None of these children required treatment with thyroxine. In total, 2 of 276 children (0.7%) had subclinical hyperthyroidism (TSH, 0.31–0.33 mIU/L) after three months.

#### 3.2.2. ESS

At diagnosis, none of the children had ESS. After three months, 1.5% (4/265) of children had developed ESS. In 33% (49/148) of children, an isolated rT3 elevation was found at diagnosis (median rT3 concentration of 0.25 ng/mL (range of 0.22–0.58)) which increased to 50% (133/265) after three months (median of 0.27 ng/mL (range of 0.22–2.36)). A significant, weak, positive correlation was found between the FT4 and rT3 concentrations three months after diagnosis (r = 0.18, 95% CI 0.06–0.29).

Children with an isolated elevated rT3 concentration after three months were slightly younger (7.7 compared with 9.6 years), more frequently had a brain tumor (74% versus 48%; *p* = 0.009) and were less often treated with anthracyclines (65% versus 80%; *p* = 0.006) than those without. No associations were found between corticosteroid use <48 h earlier or physical condition and having elevated rT3. In multivariable analysis, brain tumor diagnosis was the only significant risk factor for developing an elevated rT3 concentration three months after diagnosis (OR 3.17, 95% CI 1.19 to 8.41) (Table 3).

#### 3.2.3. Central Hypothyroidism

After three months, 1.9% (5/265) of children were suspected of having central hypothyroidism with lowered FT4 (median FT4 of 8 pmol/L (range of 8–9)), non-elevated TSH (median TSH of 2.80 mIU/L (range of 1.80–4.00)) and non-elevated rT3 concentrations (median of 0.17 ng/mL (range of 0.11–0.20)). All five had been diagnosed with leukemia at a median age of 5.4 years (range of 4.4–13.4). None was started on thyroxine treatment, but the thyroid profile was followed over time.

### 3.3. Decline in FT4 over Time

**Figure 2:**
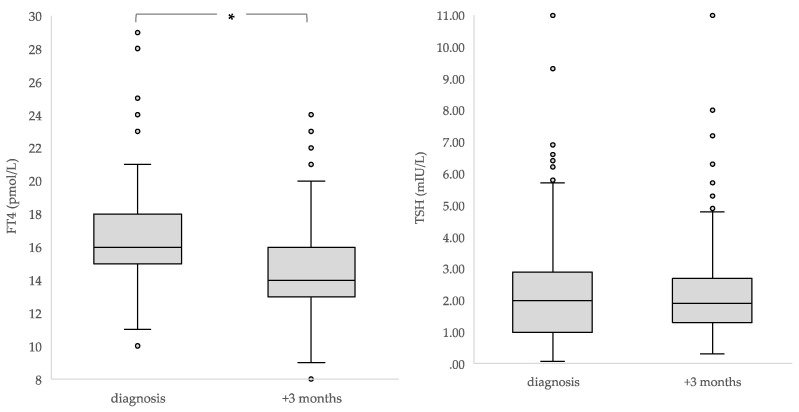

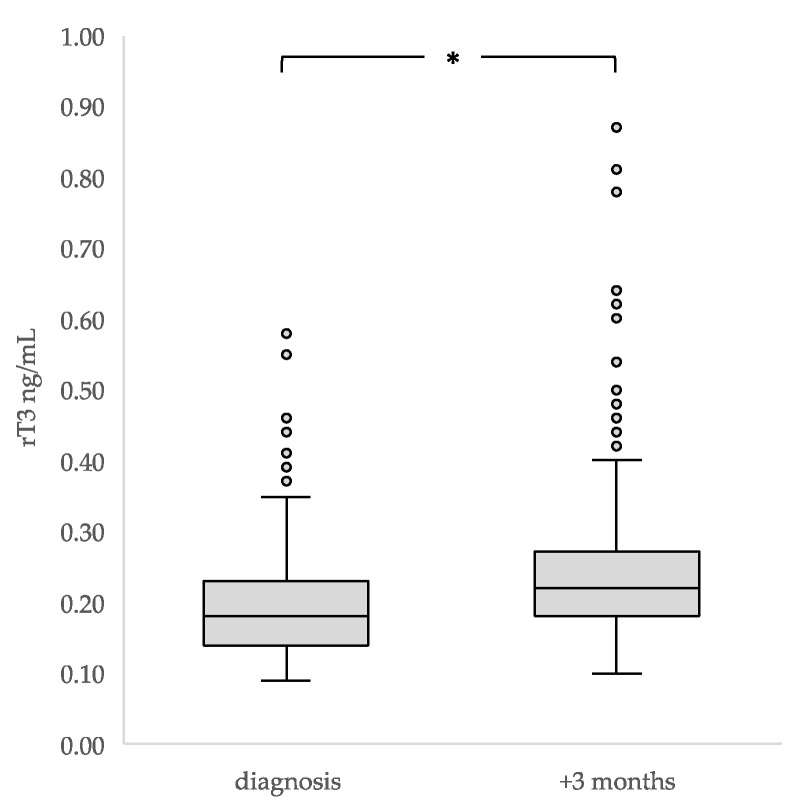
Median concentrations and interquartile ranges of TSH, FT4 and rT3 in children at diagnosis and three months after diagnosis (*n* = 284). * The boxes show the IQR divided by the median, dots denote outliers.

Overall, the median FT4 concentration declined significantly in three months’ time from a median of 16 to 14 pmol/l (*p* < 0.001), with no change in TSH (*p* = 0.334). Median rT3 concentrations significantly increased (0.18 versus 0.22 ng/ml; *p* < 0.001) (Table 2, Figure 2).

At time of diagnosis, 29% (82/284) of children had received corticosteroids <48 h earlier or chemotherapy before the first measurement. In this group, at diagnosis, lower median TSH and a higher median FT4 concentration were found when compared with those who had not (TSH, 1.20 (range of 0.07–11.00) versus 2.30 mIU/L (range of 0.34–9.40); *p* < 0.001; FT4, 17 (range of 11–28) versus 16 pmol/L (range of 10–29); *p* = 0.017). In the 22 children who had received corticosteroids <48 h before the blood withdrawal after three months, no differences were found in either TSH or FT4 concentration. (Supplementary File S2).

Due to the differences found in median plasma TSH and FT4 concentrations in the children who had already received corticosteroids <48 h earlier or chemotherapy before their first thyroid hormone measurement at diagnosis, these children were excluded from the analysis of the changes in thyroid function over time. TSH and FT4 concentrations were found to significantly decline in three months’ time (median TSH from 2.35 to 1.90 mIU/L; *p* < 0.001; median FT4 from 16 to 14 pmol/L; *p* < 0.001). The median rT3 concentrations increased significantly (0.16 to 0.22 ng/ml; *p* < 0.001) (Table 2).

The median overall change in FT4 concentration in children who had not received corticosteroids <48 h earlier or chemotherapy before the first measurement was −11% (range of −47% to +100%). FT4 declines of ≥10%, ≥20% and ≥30% were found in 41% (69/136), 28% (38/136) and 7.4% (10/136) of children, respectively.

In children with a FT4 decline of ≥20%, the median FT4 concentration declined from 17 (range of 10–29) to 12 pmol/L (range of 8–16), with no changes in median TSH and rT3 concentrations. Of these children, 36.1% had an elevated rT3 concentration after three months. The univariate analysis showed that children with a ≥20% FT4 decline were of similar age (7.7 ± 5.1 years versus 10.0 ± 5.7; *p* = 0.200), more often received antimetabolites (84% versus 67%; *p* = 0.049)) and showed a trend towards more frequent treatment with vinca-alkaloids (92% versus 80%; *p* = 0.081) compared with those with no decline or a decline of <20%. The multivariable analysis, however, did not show risk factors for a ≥20% FT4 decline (Table 3). No clinically significant effect of a ≥20% FT4 decline from baseline on BMI SDS or linear growth was found.

### 3.4. Radiotherapy

Radiotherapy was given to 21 (7.4%) children in the three months, in seven children possibly including the thyroid gland, and in 20 children, possibly including the hypothalamic–pituitary region in the radiation field. In total, 18 of the 21 children were irradiated for a brain tumor, of which 7 were craniospinal tumors (medulloblastoma, *n* = 5 (total dose of 54.0 Gray), and ependymoma, *n* = 2 (total dose of 59.4 Gray)) and 11 were cranial tumors (high-grade glioma, *n* = 10 (total dose 13–60 Gy), and germ-cell tumor, *n* = 1 (total dose 40.0 Gray)). Three children were irradiated for a sarcoma (2/3 orbit, total dose of 45–50 Gray). Median FT4 in children with radiotherapy changed from 15 (range of 13–24) to 14 pmol/L (range of 8–23) (*p* = 0.034), while median TSH remained unchanged. Reverse T3 concentrations after three months were significantly higher in children who had received radiotherapy than those in children who had not (0.28 (range of 0.14–0.62) and 0.21 ng/mL (range of 0.10–2.36); *p* = 0.015).

## 4. Discussion

In this large prospective study investigating the percentage and severity of thyroid dysfunction in children treated for newly diagnosed cancer, we found a low percentage of (subclinical) hypo- and hyperthyroidism in the first three months after starting treatment, which may be considered reassuring. In addition, the percentage of children that developed ESS, in this study defined as having lowered FT4, normal TSH and increased rT3, was low. However, in a considerable percentage of children, the thyroid profile was found to have changed, with an individual decline in FT4 concentration of ≥20% in 28% of children after three months. We did not detect clinical consequences of this change in FT4 in this relative short period of time, and future studies are needed with prolonged follow-ups.

Based on these results, we suggest that with the current treatment protocols, surveillance for hypo- and hyperthyroidism is unnecessary at this stage of treatment. However, our results do illustrate that the thyroid profile can severely change during cancer treatment in children, which may reflect adaptation to an altered metabolic state during illness or may be iatrogenic [16,17,18].

In ESS, the adaptive downregulation of TRH secretion may result in low-to-normal TSH concentrations with lowered thyroid hormone concentrations. Apart from this, in ESS, the alteration of liver deiodinases decreases the conversion of T4 into T3 and increases the conversion of T4 into rT3. In case of doubt between central hypothyroidism or ESS, the determination of rT3 may be used to differentiate them, as in true central hypothyroidism, rT3 is low, while in ESS, this is increased. The high percentage of isolated elevated rT3 concentration in our cohort may thus illustrate the presence of (mild) ESS, which may not be surprising, as these children undergo intensive treatment [19]. We could not correlate the rT3 increase to corticosteroid use, although 90% of children had received different kinds of corticosteroids within the three months.

Brain tumor diagnosis was found to be a risk factor for elevated rT3. Although no associations were found among poor physical state, corticosteroids and elevated rT3, it must be considered that brain tumor patients may have been in worse physical state compared with others, amongst others caused by cranial radiotherapy. No central hypothyroidism was found, as expected, because radiotherapy is unlikely to cause pituitary dysfunction after such a short period of time [20].

Van Iersel et al. showed that an FT4 decline of >20% during prolonged follow-up, although within reference ranges, was associated with weight gain, reduced linear growth and less improvement of intelligence scores over time in childhood brain tumor survivors [14]. This FT4 decline was regarded as a reflection of mild central hypothyroidism. Even though the etiology of declining FT4 as result of mild central hypothyroidism and (mild) ESS may not be comparable, we hypothesize that prolonged lowered thyroid hormone concentrations in (non-acutely ill) children with cancer may contribute to adverse late effects, such as short stature, weight gain, dyslipidemia, fatigue or the pathogenesis of early frailty, on childhood cancer survivors [14,21,22,23]. Therefore, we aim to follow thyroid hormone parameters in relation to these possible adverse late effects until the end of cancer treatment in this large prospective cohort.

It is not recommended to treat children with thyroid hormone for ESS during acute illness [13]. When FT4 declines in time and remains lowered for a prolonged period in “chronically” ill children, this disease state may, however, be compared to adaptation of the hypothalamic–pituitary axes, which is also encountered in children with other chronic diseases. Examples of such diseases are cystic fibrosis or chronic kidney disease, whereby affected children develop low insulin-like growth factor-1 concentrations or delayed puberty due chronical illness [24,25]. In these situations, treatment with sex steroids or growth hormone to improve bone development and final height are considered [26,27]. With this in mind, thyroid hormone treatment might be beneficial in the situation of prolonged lowered thyroid hormones in children with chronic illness or prolonged disease. This question needs to be addressed in future studies.

Our study also has several limitations. Firstly, the results might not be applicable to all children with cancer, because for this study, we only included children treated for leukemia, lymphoma, sarcoma or a non-pituitary brain tumor. Future studies may be performed to investigate changes in the thyroid profile in children with other types of childhood cancer. Secondly, although we aimed to measure the thyroid profile before any drugs had been administered, 29% of the children had already received corticosteroids <48 h earlier or chemotherapy before the first thyroid hormone measurement. For optimal analysis, we, therefore, excluded these children from analysis on changes in TSH and FT4 concentrations. Moreover, data on physical condition were scored by the researchers in three categories, based on the notes of the health care provider in the electronic patient chart, which may be considered a subjective way of physical condition scoring and thus a limitation.

## 5. Conclusions

Children with cancer, treated within current treatment protocols, do not seem to be at risk of hypo- and hyperthyroidism in the first three months of cancer treatment. In 28% of children, however, the median FT4 concentration significantly decreased during cancer treatment. The long-term clinical consequences thereof have to be investigated in future studies.

## Figures and Tables

**Table 1:** Baseline patient characteristics (*n* = 284).

Characteristic	
Age at diagnosis (yrs) (median, range)	9.4 (0.0–19.7)
No. of females (%)	127/284 (45%)
Diagnosis (number (% of entire cohort))	
Leukemia	141 (50%)
ALL	118 (42%)
AML	20 (7.0%)
CML	2 (0.7%)
Other	1 (0.4%)
Lymphoma	74 (26%)
Hodgkin lymphoma	35 (12%)
B-NHL/B-ALL	25 (8.8%)
Non-B NHL	12 (4.2%)
ALCL	2 (0.7%)
Sarcoma	38 (13%)
Bone tumor (osteosarcoma/Ewing sarcoma)	22 (7.7%)
Rhabdomyosarcoma	11 (3.9%)
Non-rhabdomyosarcoma	4 (1.4%)
Other	1 (0.4%)
Brain tumor	31 (11%)
ATRT	1 (0.4%)
Ependymoma	5 (1.8%)
Low-grade glioma	3 (1.0%)
High-grade glioma	10 (3.5%)
Germ-cell tumor	2 (0.7%)
Medulloblastoma	9 (3.2%)
Optic glioma	1 (0.4%)
Physical condition *	
At diagnosis	
Good	76 (38%)
Medium	112 (57%)
Poor	10 (5.1%)
*Unknown*	22
After three months	
Good	186 (69%)
Medium	74 (28%)
Poor	8 (3.0%)
*Unknown*	8

Abbreviations: ALL, acute lymphoblastic leukemia; AML, acute myelogenous leukemia; CML, chronic myelogenous leukemia; B-NHL, B-cell non-Hodgkin lymphoma; B-ALL, B-cell acute lymphoblastic leukemia; non-B-cell non-Hodgkin lymphoma; ALCL, anaplastic large-cell lymphoma; ATRT, atypical teratoid rhabdoid tumor. * Physical condition was scored as “good” (no complaints), “medium” (moderate complaints, “not feeling well” or “feeling tired”) or “poor” (severe complaints or “feeling ill”) as reported by the health care provider in the electronic patient chart.

**Table 2:** Median plasma concentration of thyroid determinants in children with cancer measured at diagnosis and three months after diagnosis.

	All Children (*n* = 284)			Analysis of Children Who Had Not Received Corticosteroids or Chemotherapy before First Measurement Only (*n* = 202)		
Thyroid Hormone Determinant	Median Concentration at Diagnosis, mIU/L (Range) (No. of Samples)	Median Concentration Three Months after Diagnosis, mIU/L (Range) (No. of Samples)	*p*-Value	Median Concentration at Diagnosis before Start of Chemotherapy or Corticosteroids, mIU/L (Range) (No. of Samples)	Median Concentration Three Months after Diagnosis, mIU/L (Range) (No. of Samples)	*p*-Value
TSH (0.30–5.00 mIU/L)	2.00 (0.07–11.0) (*n* = 222)	1.90 (0.31–8.00) (*n* = 276)	0.334	2.30 (0.34–9.40) (*n* = 141)	1.90 (0.31–11.00) (*n* = 199)	<0.001
FT4 (10–22 pmol/L) *	16 (10–29) (*n* = 220)	14 (8–23) (*n* = 276)	<0.001	16 (10–29) (*n* = 139)	14 (8–24) (*n* = 199)	<0.001
rT3 (0.098–0.218 ng/mL)	0.18 (0.09–0.58) (*n* = 148)	0.22 (0.10–2.36) (*n* = 265)	<0.001	0.16 (0.09–0.58) (*n* = 90)	0.22 (0.10–2.26) (*n* = 191)	<0.001

Abbreviations: TSH, thyroid stimulating hormone; FT4, free thyroid hormone; rT3, reverse T3. * Dependent on age: 20 days–3 years, 12–21 pmol/L; 3–5 years, 10–19 pmol/L; 5–19 years, 11–20 pmol/L; >19 years, 10–22 pmol/L. *p*-Value: for analysis of changes in thyroid hormone concentrations, only paired blood samples (diagnosis and three months after diagnosis) were used.

**Table 3:** Risk factor analysis results.

**A. Risk Factors Associated with ≥20% FT4 Decline**		
**Covariate/Category**	**≥20% FT4 Decline**	
	**Univariable** **OR (95% CI)**	**Multivariable** **OR (95% CI)**
Age at diagnosis, years	0.95 (0.89 to 1.02)	0.97 (0.90 to 1.04)
Administration of antimetabolites	2.59 (0.98 to 6.81)	2.37 (0.78 to 7.18)
Use of corticosteroids <48 h before thyroid hormone measurement after three months	1.11 (0.27 to 4.55)	1.55 (0.35 to 6.97)
Radiotherapy before thyroid hormone measurement after three months	0.54 (0.11 to 2.67)	0.93 (0.15 to 5.65)
**B. Risk factors associated with elevated rT3 concentration**		
**Covariate/Category**	**Elevated rT3 Concentration**	
	**Univariable** **OR (95% CI)**	**Multivariable** **OR (95% CI)**
Age at diagnosis, years	0.96 (0.92 to 1.00)	0.97 (0.92 to 1.01)
Brain tumor vs. others ^a^	3.16 (1.29 to 7.76)	3.17 (1.19 to 8.41) ^b^
Physical condition three months after diagnosis Medium/poor vs. good	1.54 (0.90 to 2.64)	1.42 (0.80 to 2.53)
Use of corticosteroids <48 h before thyroid hormone measurement	1.68 (0.67 to 4.20)	1.72 (0.67 to 4.43)
Underweight (<−2 SDS)	0.51 (0.12 to 2.08)	0.57 (0.13 to 2.45)
NOTE. 3A. Multivariable logistic regression for risk factors of children with a ≥20% FT4 decline from diagnosis to three months after diagnosis (*n* = 38) compared with children without ≥20% FT4 decline from diagnosis to three months after diagnosis (*n* = 98). NOTE. 3B. Multivariable logistic regression for risk factors of children with elevated rT3 concentrations three months after diagnosis (*n* = 133) compared with children without elevated rT3 concentrations three months after diagnosis (*n* = 132).		

Abbreviations: FT4, free thyroxine; OR, odds ratio; CI, confidence interval; SDS, standard deviation score. ^a^ Brain tumor diagnosis versus other diagnoses. ^b^ Statistically significant.

## Data Availability

The datasets used and/or analyzed during the current study are available from the corresponding author upon reasonable request.

## Supplementary Materials

The following supporting information can be downloaded at: https://www.mdpi.com/article/10.3390/cancers15051500/s1, File S1: Laboratory assays; File S2: Chemotherapeutic Agents and Corticosteroids.
