# Supplementary material for: Thyroid Profile in the First Three Months after Starting Treatment in Children with Newly Diagnosed Cancer
Source: Cancers (Basel). 2023 Feb 27;15(5):1500. doi: 10.3390/cancers15051500 (PMC10000403; doi:10.3390/cancers15051500)
Supplement: Supplementary file 1 [file cancers-15-01500-s001.zip › cancers-2232715 - Supplementary File S1.pdf]

Supplementary Files

Supplementary File S1. Laboratory assays

FT4 and TSH concentrations were measured using the Atellica IM analyzer ®, (Siemens Healthcare Diagnostics Inc., Erlagen, Germany). Serum reverse T3 was measured using an Enzyme-Linked Immunosorbent Assay (ELISA) kit (TECAN IBL International, Hamburg, Germany). For determination of antibodies against thyroid peroxidase (anti-TPO) the 'Phadia250' immunoassay analyzer was used (Thermo Fisher Scientific Inc., Waltham, United States).
