# Supplementary material for: Thyroid Profile in the First Three Months after Starting Treatment in Children with Newly Diagnosed Cancer
Source: Cancers (Basel). 2023 Feb 27;15(5):1500. doi: 10.3390/cancers15051500 (PMC10000403; doi:10.3390/cancers15051500)
Supplement: Supplementary file 1 [file cancers-15-01500-s001.zip › cancers-2232715 - Supplementary File S2.pdf]

Supplementary File S2. Chemotherapeutic Agents and Corticosteroids

A. ***Overview of groups of chemotherapeutic agents and corticosteroids administered in children three months after diagnosis.***

**Chemotherapy groups**	**Three months after diagnosis**	
	N=276	
	Total	<7 days
	N (%)	N (%)
Alkylating agents	216 (79)	16 (5.8)
Antimetabolites	192 (70)	34 (12)
Anthracycline antibiotics	197 (71)	3 (1.1)
Asparaginase	128 (46)	19 (6.9)
Oncolytica	4 (1.4)	2 (0.7)
Protein kinase inhibitors	8 (2.9)	7 (2.5)
Topo-isomerase inhibitors	86 (31)	1 (0.4)
Vinca-alkaloids	235 (85)	18 (6.5)
**Corticosteroids**	Total	<48 hours
	N (%)	N (%)
Treatment protocol	243 (89)	2 (0.7)
Other^	5 (1.8)	20 (7.2)

Chemotherapeutic agents or corticosteroids administered three months after diagnosis.
^Including corticosteroids used as supportive care drugs e.g. as anti-emetic drugs.

B. ***Group Classification of Chemotherapeutic Agents and Corticosteroids***

Alkylating agents: Cyclofosfamide, Dacarbazine, Ifosfamide. Lomustine, Thiotepa, Busulfan, Temozolomide, Melfalan, Bendamustine, Carmustine, Treosulfan
Antimetabolites: **Cytarabine,** Fludarabine, Fluorouracil, Mercaptopurine, Methotrexaat, Tioguanine, Azacitidine, Clofarabine, Gemcitabine, Nelarabine 5 mg/ml
Anthracycline antibiotics: Bleomycine, Dactinomycine, Daunorubicine, Doxorubicine, Mitoxantrone, Daunorubicin+Cytarabin, Daunorubicine, Idarubicine
Asparaginase: Pegasparaginase (Oncaspar), Spectrila, Erwinase, Asparaginase, E.Coli (Spectrila) Asparaginase, Erwinia (Crisantaspase)
Oncolytica: Arseentrioxide
Protein kinase inhibitor: Bortezomib, Imatinib, Quizartinib, Brigatinib, Dasatinib, Ruxolitinib, Ponatinib, Crizotinib
Topo-isomerase inhibitor: Etoposide, Irinotecan, Topotecan
Vinca-alkaloids: Vinblastine, Vincristine, Vindesine, Vinorelbine
Corticosteroids: Dexamethason, Methylprednisolon, Prednisolon, Hydrocortison
